# Establishment and Characterization of a Novel Rat Model of Mechanical Low Back Pain Using Behavioral, Pharmacologic and Histologic Methods

**DOI:** 10.3389/fphar.2017.00493

**Published:** 2017-07-27

**Authors:** Arjun Muralidharan, Thomas S. W. Park, John T. Mackie, Luiz G. S. Gimenez, Andy Kuo, Janet R. Nicholson, Laura Corradini, Maree T. Smith

**Affiliations:** ^1^Centre for Integrated Preclinical Drug Development, The University of Queensland, Brisbane QLD, Australia; ^2^School of Veterinary Science, The University of Queensland, Gatton QLD, Australia; ^3^Boehringer Ingelheim Pharma GmbH & Co. KG Biberach, Germany; ^4^School of Pharmacy, The University of Queensland, Brisbane QLD, Australia

**Keywords:** mechanical low back pain (LBP), intervertebral disks (IVD), mechanical allodynia, algometry, gait, gabapentin, IVD histology, rat model of LBP

## Abstract

Chronic low back pain (LBP), the leading cause of disability globally, is notoriously difficult to treat. Most rodent models of LBP mimic lumbar radicular pain rather than mechanical LBP. Here, we describe establishment of a new rat model of mechanical LBP that is devoid of a neuropathic component. Groups of adult male Sprague Dawley rats were anesthetized and their lumbar L4/L5 and L5/L6 intervertebral disks (IVDs) were punctured (0.5 mm outer diameter, 2mm-deep) 5 (LPB-5X), or 10 (LBP-10X) times per disk. Sham-rats underwent similar surgery, but without disk puncture. Baseline noxious pressure hyperalgesia of lumbar axial deep tissues, mechanical allodynia in the hindpaws and gait were assessed prior to surgery and once-weekly until study completion on day 49. The model was also characterized using pharmacologic and histologic methods. Good animal health was maintained for ≥ 49 days post-surgery. For LBP- but not sham-rats, there was temporal development of noxious pressure hyperalgesia in lumbar axial deep tissues at days 14–49 post-surgery. Importantly, there were no between-group differences in von Frey paw withdrawal thresholds or gait parameters until study completion. On day 49, significant histologic changes were observed in the L4/L5 and L5/L6 IVDs for LBP-10X rats, but not sham-rats. In LBP-10X rats, single bolus doses of morphine produced dose-dependent relief of primary and secondary mechanical hyperalgesia in lumbar axial deep tissues at L4/L5 and L1, respectively. In conclusion, our new rat model has considerable potential for providing novel insight on the pathobiology of mechanical LBP and for analgesic efficacy assessment of novel compounds.

## Introduction

Globally, chronic low back pain (LBP) is a leading cause of disability that is notoriously challenging to treat (Hsu et al., 2015). In the adult population, chronic LBP ranked first for disability of 291 conditions in the Global Burden of Disease 2010 study (Hoy et al., 2014) and sixth in terms of overall socioeconomic burden in disability-adjusted life years (Murray et al., 2012). Additionally, chronic LBP has a global point prevalence of 9.4% and a lifetime prevalence of 50–90% (Hoy et al., 2014; Hsu et al., 2015). Currently available drug treatments for the relief of chronic LBP are paracetamol, non-steroidal anti-inflammatory drugs (NSAIDs), corticosteroids and opioids (Hsu et al., 2015). However, these medications only provide partial pain relief and are associated with considerable side-effects (Hsu et al., 2015). As a result, poorly relieved chronic LBP not only causes enormous patient disability and suffering, but also a high socioeconomic burden on the affected individuals, their families, industry and Governments, due to work days lost and incurred healthcare costs (Forster et al., 2013; Hsu et al., 2015). Hence, chronic LBP is a large unmet medical need (Peng, 2013) demanding discovery and development of highly effective and well-tolerated novel analgesics (Forster et al., 2013).

Despite the high incidence and prevalence of chronic LBP, the precise pathobiological mechanisms that underpin its development remain largely unknown. However, the source of LBP is often associated with intervertebral disks (IVDs), facet and/or sacroiliac joints, with discogenic LBP more common in adults ≤ 70 years (DePalma et al., 2011). Mechanical LBP, due to noxious stimulation of structures in the lumbar spine is described as a type of nociceptive pain (Bogduk, 2009). By contrast, lumbar radicular pain due to disk herniation and pressure on nearby nerve roots accounts for < 12% of LBP cases in the clinical setting (Bogduk, 2009).

Multiple rodent models of chronic LBP have been used to investigate the mechanisms underpinning the degeneration of IVDs due to age-related changes (Gruber et al., 2002), injury-induced by mechanical (Masuda et al., 2005; Rousseau et al., 2007; Olmarker, 2008; Kim et al., 2011) or chemical methods (Aoki et al., 2004a,b), or due to genetic inactivation of specific IVD proteins (Boyd et al., 2008; Millecamps et al., 2012). Amongst these models, the most widely used method for inducing chronic LBP in rodents is the IVD puncture model as it yields a progressive and reproducible degeneration, similar to the changes observed in humans (Daly et al., 2016). Although few studies have described the associated pain behavioral phenotypes (Olmarker, 2008; Kim et al., 2011; Henry et al., 2012; Li et al., 2014; Lai et al., 2015, 2016), a significant limitation of many previous models is that they mimic lumbar radicular pain more closely than chronic mechanical (discogenic) LBP in humans (Devor and Tal, 2009; Defrin et al., 2014). This is in part due to the removal and/or leakage of nucleus pulposus (NP) from the IVDs in these rodent models and their possible exposure to the adjacent sensory nerve roots, resulting in lumbar radicular pain (Takahashi et al., 2008). Hence, our aims were to establish and characterize a new rat model of chronic mechanical LBP that involves shallow annular punctures to the lumbar IVDs, without removal of the NP, thereby preventing the concurrent development of a lumbar radicular pain component. Such a model would have application for generating novel insight on the pathobiology of this condition. It will also facilitate efficacy profiling of compounds from drug discovery to identify those with potential to be progressed into development as novel analgesics for improved relief of discogenic pain in humans.

Briefly, we compared the impact of either 5 or 10 shallow puncture wounds (0.5 mm outer diameter (o.d.), 2 mm deep) in the lumbar L4/L5 and L5/L6 IVDs of anesthetized rats, on temporal development of mechanical hypersensitivity in lumbar axial deep tissues at L4/L5 (primary hyperalgesia) and L1 (secondary hyperalgesia) over a 49-day study period. We characterized our new rat model of LBP using gait analysis, histology and pharmacological profiling with morphine, gabapentin, amitriptyline, and meloxicam.

## Materials and Methods

### Drugs and Reagents

Topical antibiotic powder and pentobarbitone sodium (Lethabarb^®^) were purchased from Apex Laboratories Pty Ltd (Somersby, NSW, Australia) and Virbac (Australia) Pty Ltd (Penrith, NSW, Australia), respectively. Medical grade O_2_ and CO_2_ were purchased from Coregas (Brisbane, QLD, Australia). Gabapentin was provided by Dr Ben Ross, School of Pharmacy, The University of Queensland (Brisbane, QLD, Australia). Buprenorphine hydrochloride (Temgesic^®^) was purchased from Reckitt Benckiser (Australia) Pty Ltd (Sydney, NSW, Australia).

### Animals

This study was conducted in accordance with the guidelines set out in the Australian Code of Practice for the Care and Use of Animals for Scientific Purposes (8th Edition, 2013) (NHMRC, 2013). All experiments adhered to the guidelines of the Committee for Research and Ethical Issues of the International Association for the Study of Pain (IASP). Animal ethics approval was obtained from the Animal Ethics Committee of The University of Queensland prior to study commencement.

Adult male Sprague-Dawley (SD; 180–200 g) rats were purchased from the Animal Resources Centre (Perth, WA, Australia). They were housed upon arrival in groups of two to three in a temperature-controlled room (21°C ± 2°C) in the animal holding facility equipped with a 12 h/12 h light-dark cycle. Environmental enrichment comprised placement of rodent hutches and rat chew sticks in all home cages. Standard rodent chow and water were available *ad libitum*. Rats were acclimatized for at least 3 days prior to initiation of experimentation.

### Surgical Procedure

Male Sprague-Dawley rats (200–225g) were administered an intraperitoneal (i.p.) injection comprising xylazine (8 mg/kg) in a 1:1 combination with tiletamine-hydrochloride and zolazepam-hydrochloride (Zoletil^®^; 1:1; 50 mg/kg). Once deeply anesthetized, rats underwent a surgical procedure and annular puncture of the L4/L5 and L5/L6 IVDs, as previously described (Kim et al., 2011) but with an important modification such that the NP remained intact in contrast to its removal by others (Kim et al., 2011). Briefly, a midline ventral abdominal incision was made and the abdominal viscera were gently retracted to access the lumbar disk space. The viscera were irrigated with sterile saline solution and covered with moist sterile gauze to maintain tissue hydration. Next, the lumbar L4/L5 and L5/L6 IVDs were punctured 5 (LBP-5X) or 10 (LBP-10X) times per disk to a depth of 2mm using a 25G needle (0.5 mm o.d.) with a polyethylene sleeve cut 2 mm shorter than the length of the needle. After completion of the disk puncture procedure, the muscle and skin were closed in layers using 4-0 sterile silk sutures, and the animals were kept warm and monitored closely during post-surgical recovery for at least 3 h. Once rats regained consciousness, they were housed individually for at least 2 days post-surgery to allow wound to close. Thereafter, they were housed in groups of 2 to 3 per cage. General animal health and body weights were assessed once-weekly until the end of the experimental period at 49-days post-surgery. On the day of surgery and on the first post-operative day, rats received subcutaneous buprenorphine at 0.1 mg/kg for post-operative analgesia. Sham-rats underwent the same surgical procedure to expose but not puncture the lumbar IVDs. A separate group of age-matched procedure-naïve rats was also included for comparative purposes in the model characterization.

### Behavioral Studies

Each experimental cohort comprised LBP-rats (LBP-5X and/or LBP-10X) and sham-rats. To fully characterize the model, some cohorts also included a group of age-matched, non-operated control animals. The total number of rats per cohort and the behavioral experiments performed are summarized in **Table 1**.

**Table 1:** Details of group sizes for all pain behavioral and gait analysis experiments.

Cohorts	Number of animals in each group				Experiments performed^¶^
	Naïve	Sham	LBP-5X	LBP-10X	
1	2	3	4	–	PAT only at L4/L5
2	3	3	6	–	PAT only at L1
3	3	3	6	–	PAT at both L1 & L4/L5
4	–	3	4	5	PAT at both L1 & L4/L5
5	–	3	–	9	PAT at both L1 & L4/L5
6	4	4	–	4	Gait analysis using Catwalk^TM^ and PAT at both L1 & L4/L5

*^¶^Paw withdrawal thresholds (PWTs) were assessed on both left and right hindpaws for all cohorts. PAT, pressure algometry thresholds.*

#### Pressure Algometry

The sensitivity of lumbar axial deep tissues to applied noxious mechanical force was measured using an algometer (Ugo Basile, Italy) in *Experimental Cohort 1 – 6 rats*, as previously described (Kim et al., 2011). Briefly, mechanical force was applied at a rate of approximately 100 g/s on the dorsum at lumbar levels L1 and L4/L5 to assess the pressure algometry thresholds (PATs) that elicited audible vocalization or discomfort. A 1000 g cut-off was used to prevent tissue damage. The baseline PATs for L1 and L4/L5 were the mean of three readings for each region, with consecutive measurements separated by at least 5-min intervals. Algometry assessments were performed prior to surgery and then once-weekly until study completion at 49-days post-surgery. Multiple testers performed the pressure algometry measurements on the various days to minimize the potential for experimenter bias. Moreover, on each testing occasion, animals from different experimental groups (i.e., LBP, sham and naive) were pooled together in a large cage, and the baseline values were noted against the numbers shown on the animal tail. This was done to ensure that the experimenter remained *‘blinded’* to the experimental group allocation during the testing period. Pressure hyperalgesia was considered to be developed in LBP-rats when the mean PATs were ≥ 100 g lower than the corresponding baseline values of sham and/or age-matched control non-operated rats. On a particular note, the differences in the ‘n’ numbers presented in **Figures 3A,B** are because ‘*Cohort 1*’ rats had PATs assessed only in the L4/L5 region, whereas for ‘*Cohort 2*’ rats, PATs were assessed only in the L1 region (**Table 1**).

#### Assessment of Mechanical Allodynia in the Hindpaws

Calibrated Semmes-Weinstein von Frey filaments (Stoelting Co., Wood Dale, IL, United States) were used to determine the lowest mechanical threshold required to evoke a brisk hindpaw withdrawal reflex in *Experimental Cohort 1 – 6* rats, as previously described (Muralidharan et al., 2013). Briefly, rats were placed individually into wire mesh testing cages and allowed to acclimatize for 15–20 min prior to testing. Commencing with the 6 g filament, the filament was applied to the plantar surface of the hindpaw until it buckled slightly. Absence of a response after 3 s prompted use of the next filament of increasing force. In contrast, the observation of a hindpaw withdrawal response within 3 s prompted use of the next filament of decreasing force (Muralidharan et al., 2013). A score of 20 g was given to rats that did not respond to any of the von Frey filaments in the range 2–20 g. The baseline paw withdrawal thresholds (PWTs) for each of the left and right hindpaws were the mean of three readings for each hindpaw with a 5-min interval between consecutive measurements. Mechanical allodynia was considered to be fully-developed in the hindpaws when the mean PWTs were ≤ 6 g. The bilateral hindpaw PWTs were assessed prior to surgery and thereafter at once-weekly intervals until study completion at day 49 post-surgery.

#### Gait Analysis

Possible temporal changes in the gait of rats in *Experimental Cohort 6* were assessed using a CatWalk^TM^ XT system (version 10.5; Noldus Information Technology; Wageningen, Netherlands), as previously described (Vrinten and Hamers, 2003). Briefly, all animals were habituated and trained to ambulate on the CatWalk^TM^ XT system on three successive days prior to measurement of their baseline responses. On each individual training day (i.e., days -3, -2, and -1), rats were removed from their home cage and were placed at the end of a covered illuminated glass walkway (160 cm × 20 cm). The CatWalk^TM^ experiments were performed in a dark room with the only light sources being that of the CatWalk^TM^ instrument itself and that of a computer monitor (Dell; 24inch) facing away from the CatWalk^TM^ platform. The home cage containing cage-mates was placed at the opposite end of the CatWalk^TM^ walkway as an attractant. Progression of each animal along the walkway to its home cage was captured using a high-speed camera located at 70 cm beneath the walkway. This process was repeated until three good runs were obtained (i.e., smooth and constant unidirectional movement). For detection, the gain and green intensity threshold of the camera component of the Catwalk^TM^ Sytem were set at 11.99 and 0.10, respectively. Detection settings were determined based upon our experimental environment to achieve an optimal intensity range for the paw prints and to filter out background noise. In this study, base of support (BOS), an inter-limb coordination parameter that is a measure of the distance between the two forepaws or the two hindpaws, was analyzed. Baseline gait analysis was performed prior to LBP- or sham-surgery and then at once-weekly intervals until study completion at 49 days post-surgery.

#### Analgesic Efficacy of Clinically Available Drug Treatments for Relief of Mechanical Hyperalgesia

Dosing solutions of morphine (0.1, 0.3, and 1.0 mg/kg; s.c.), gabapentin (30, 60, and 100 mg/kg; i.p.), amitriptyline (1, 3, and 7 mg/kg; i.p.), meloxicam (3, 10, and 30 mg/kg; i.p.) and vehicle (sterile water for injection; i.p.) were prepared by one person and were assigned codes independently by a second person. The coded test solutions (morphine, gabapentin, meloxicam, amitriptyline or vehicle) were administered to rats by the first person and testing was undertaken in a *‘blinded’* manner. The assigned codes were revealed to the experimenter only after completion of testing. At days 28–40 post-surgery, separate groups of LBP-10X rats with significant mechanical hypersensitivity (≥100 g lower than the corresponding PATs for the sham-group) of the lumbar axial deep tissues at L4/L5 (primary hyperalgesia) and L1 (secondary hyperalgesia), received single bolus doses of clinically available pain relieving-agents: amitriptyline (1, 3, and 7 mg/kg; *n* = 6, 8 and 7, respectively), gabapentin (30, 60, and 100 mg/kg; *n* = 6, 6 and 7, respectively), meloxicam (3, 10, and 30 mg/kg; *n* = 6 per dose) and morphine (0.1, 0.3, and 1 mg/kg; *n* = 6 per dose), or vehicle (*n* = 14), according to a ‘2-day washout’ protocol such that there were at least 2 days between successive doses. PATs were assessed in a ‘*blinded*’ manner in the lumbar L1 region pre-dose and at 15, 30, 45, 60, 75, 90, 120, and 180 min post-dosing. Similarly, PATs were assessed in a ‘*blinded*’ manner in the lumbar L4/L5 region pre-dose and at 15, 30, 45, 60, 75, 90, 120, and 180 min post-dosing in the same rats.

### Histology

Separate groups of sham (*n* = 3) and LBP-10X (*n* = 5) rats were euthanized on day 49 post-surgery with an overdose of pentobarbitone. The lumbar L4/L5 and L5/L6 IVDs were collected from each rat as a single vertebral segment from L3 to L6. To maintain orientation, the ventral sides of each L4 vertebral segment were stained with Evans Blue dye in an arrow shape pointing toward the cranial aspect of each animal. The collected IVDs were then stored in 10% formalin for at least 24 h, followed by decalcification in 15% EDTA for at least 8 to 12 weeks. Following decalcification, the vertebrae were bisected in the sagittal plane. Subsequently, the samples were rinsed, dehydrated, embedded in paraffin and each IVD was sectioned at 4–5 μm with the L3 to L6 vertebrae visible as a whole segment. Step sections at ∼60 μm apart were collected and mounted on Superfrost^TM^ Plus slides (Thermo Fisher Scientific Inc., Waltham, MA, United States). Sections were then stained with hematoxylin and eosin (H&E) and safranin O-fast green, and the slides were scanned with an Aperio^®^ slide scanner (Leica Microsystems Pty Ltd, Springwood, NSW, Australia). The sections were evaluated in a blinded fashion by a board-certified diplomate of the American College of Veterinary Pathologists (JTM). Features evaluated included the sharpness of the boundary between the NP and the annulus fibrosus (AF) at the cranial, caudal, dorsal and ventral aspects of the IVD, disruption of the AF and degeneration of the NP. Changes were scored as 0, 1, or 2, with ‘0’ designating no abnormalities detected, ‘1’ designating mild changes and ‘2’ designating moderate or marked changes.

### Data Analyses

Pressure algometry thresholds were normalized by subtracting pre-dosing baseline values from each post-dosing PAT for each individual rat to obtain ΔPAT values. The extent and duration of pain relief [area under the ΔPAT versus time curve (ΔPAT AUC)] for each individual rat was determined using trapezoidal integration (GraphPad Prism^TM^ v7.03). These values for each rat were then normalized to the percentage of the maximum possible ΔPAT AUC (% MAX ΔPAT AUC) values as follows:

$$\begin{array}{c} \%\mathrm{MAX}\Delta \mathrm{PAT}\;AUC=\;\frac{\Delta \mathrm{PAT}\mathrm{AUC}}{\mathrm{Maximum}\Delta \mathrm{PAT}\;\mathrm{AUC}}\times \frac{100}{1} \end{array}$$

Dose-response curves were generated by plotting mean (± SEM) % MAX ΔPAT AUC values versus log morphine dose. Non-linear regression (GraphPad Prism^TM^ v7.03) was used to estimate the ED_50_ values for morphine for the relief of noxious mechanical hypersensitivity for each of the L1 and L4/L5 regions.

### Statistical Analyses

Statistical analyses were performed using repeated measures two-way analysis of variance (ANOVA) followed by the Bonferroni test to assess between-group differences in von Frey thresholds, PATs, gait parameters and body weight data. The Kruskal–Wallis non-parametric test followed by the Dunn’s test was used to compare the values between the morphine- and vehicle-treated groups of LBP-10X rats. Statistical analyses were performed using the GraphPad Prism^TM^ v7.03 program (GraphPad software) and the statistical significance criterion was *P* ≤ 0.05. For statistical comparisons using ANOVA, *F*-values together with their associated degrees of freedom are reported. Specifically, for two-way ANOVA, *F*-values are expressed as *F*_(dfoftreatment,time,interaction/residual)_.

## Results

The general animal health data and the pain behavioral data from all rat experimental cohorts have been cumulated and are described in the following sections.

### General Animal Health

**FIGURE 1:**
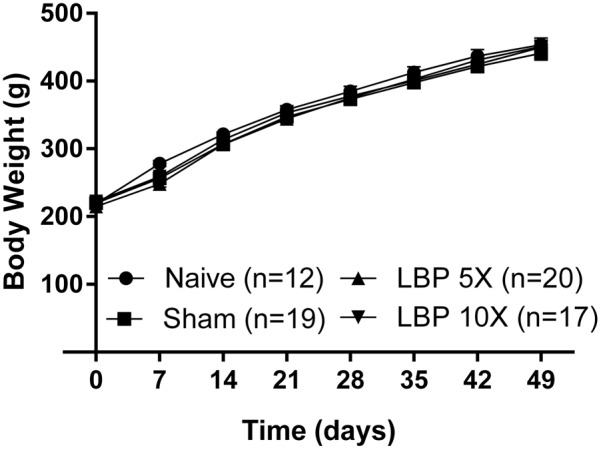
Mean (±SEM) body weights of LBP-5X (*n* = 20) and -10X (*n* = 17) rats did not differ significantly (*P* > 0.05) from those of sham- (*n* = 19) or age-matched non-operated control (*n* = 12) groups for the 49 day study period.

There were no significant [*F*_(3,7,21/64)_= 0.8, 1680, 1.3; *P* > 0.05] between-group differences in the mean (±SEM) body weights of LBP-5X and LBP-10X rats relative to sham- and age-matched non-operated groups until the end of the study period (**Figure 1**). Although there was one unanticipated rat death in the LBP-10X group (*Experimental Cohort 5*) on day 32 post-surgery, histopathologic examination by Cerberus Sciences (Adelaide, SA, Australia) found that the cause of death was thymus ablation by a lymphoblastic lymphoma, a common tumor in rats. Hence, behavioral data from this rat were excluded from the group analyses.

### Assessment of von Frey Paw Withdrawal Thresholds in the Hindpaws

**FIGURE 2:**
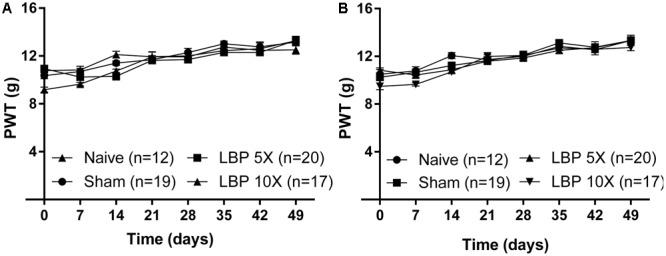
The mean (±SEM) PWT versus time curves for the **(A)** left and **(B)** right hindpaws of LBP-5X (*n* = 20) and -10X (*n* = 17) rats relative to those of sham- (*n* = 19) and age-matched non-operated control (*n* = 12) rats. There were no significant (*P* > 0.05) between-group differences in the mean (±SEM) PWTs of the left or the right hindpaws until study completion at 49-days post-surgery.

Importantly, the mean (±SEM) PWTs for the left [*F*_(3,7,21/64)_ = 2.6, 74.4, 3.2; *P* > 0.05; **Figure 2A**] and the right [*F*_(3,7,21/64)_ = 1.3, 82.2, 1.9; *P* > 0.05; **Figure 2B**] hindpaws of LBP-5X and LBP-10X rats, did not differ significantly from the corresponding values for the sham- and age-matched control groups up to 49-days post-study initiation.

### Temporal Development of Mechanical Hypersensitivity in the Lumbar Axial Deep Tissues

**FIGURE 3:**
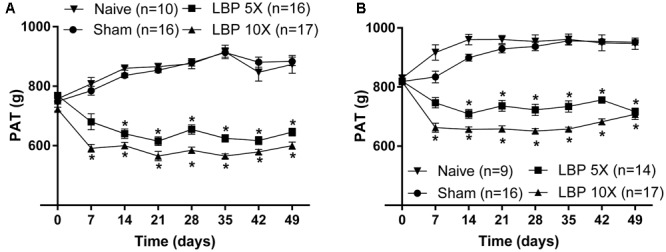
Temporal changes in the mean (±SEM) PATs for the lumbar **(A)** L1 and **(B)** L4/L5 regions of LBP-5X (*n* = 14–16) and -10X (*n* = 17) rats compared with sham- (*n* = 16) and age-matched non-operated control (*n* = 9–10) rats. At 14–49 days post-surgery, there was a significant (*P* ≤ 0.05) reduction in the mean (±SEM) PAT values determined for the lumbar axial deep tissues at both L1 and L4/L5 for LBP-5X and -10X rats *c.f.* sham- or age-matched non-operated control rats. ^∗^*P* ≤ 0.05 (Two way ANOVA, *post hoc*: Bonferroni) when compared with sham-animals.

At 14–49 days after lumbar IVD puncture, there were significant temporal decreases in the mean (±SEM) PATs for LBP-5X and LBP-10X rats, at both L1 [*F*_(3,7,21/385)_ = 322.6, 3.3, 16.01; *P* ≤ 0.05] (**Figure 3A**) and L4/L5 [*F*_(3,7,21/364)_ = 196.3, 5.3, 17.4; *P* ≤ 0.05] (**Figure 3B**), relative to the corresponding mean (±SEM) PATs for the sham- and age-matched control groups.

### Gait Changes

**FIGURE 4:**
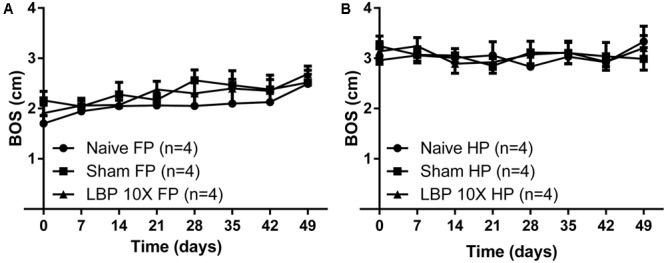
Temporal changes in the mean (±SEM) base of support (BOS) gait parameter assessed using the Catwalk^TM^system, for the **(A)** forepaws (FP) and **(B)** hindpaws (HP) of LBP-10X rats (*n* = 4) compared with sham- (*n* = 4) and age-matched non-operated control (*n* = 4) rats. There were no significant (*P* > 0.05) between-group differences in the mean ( ± SEM) BOS values (inter-limb coordination parameter) until study completion at 49-days post-surgery.

For gait assessment, the mean (±SEM) BOS versus time curves (inter-limb coordination parameter) for the forepaws and the hindpaws of LBP-10X rats relative to sham- and non-operated animals are shown in **Figures 4A,B**, respectively. There were no significant between-group differences in the mean (±SEM) BOS values for the forepaws [*F*_(2,7,14/72)_ = 3.2, 2.6, 0.27; *P* > 0.05] or the hindpaws [*F*_(2,7,14/72)_ = 0.1, 0.7, 0.4; *P* > 0.05] until completion of the study period on day 49.

### Histology

**FIGURE 5:**
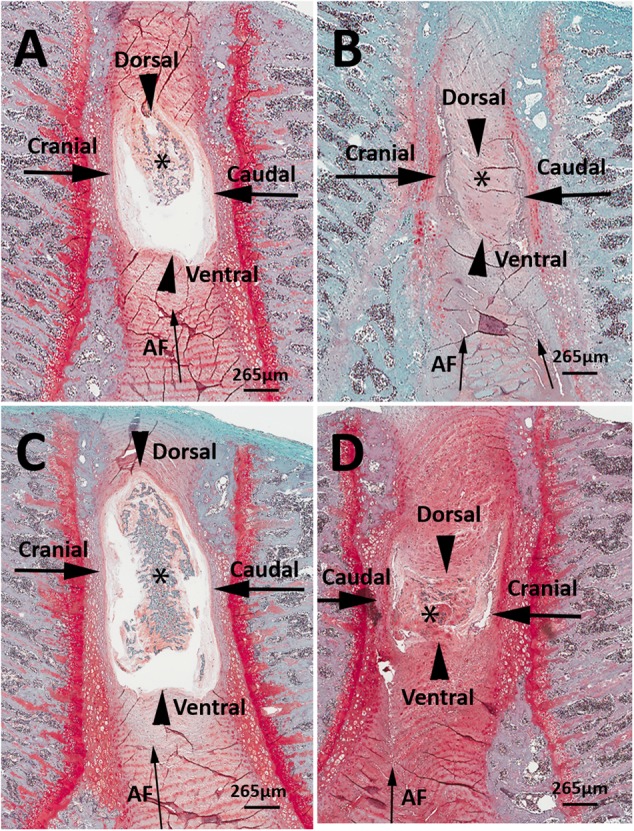
Representative histologic images of L4/5 and L5/L6 IVD sections from sham **(A,C)** and LBP-rats **(B,D)**. In comparison with sham-rats (*n* = 3), the L4/5 and L5/6 IVDs from LBP-10X rats (*n* = 5) showed loss of sharp boundaries between the nucleus pulposus (NP, asterisk) and the annulus fibrosus (AF, thin arrow) at the cranial and caudal aspects of the disk (thick arrows), as well as at the dorsal and ventral aspects of the disk (arrowheads). In addition, in LBP-10X rats, the AF was disrupted with vertical tearing and loss of orderly apposition of lamellae and there was degeneration of the NP. Safranin-O-fast green stain. Bar = 265 μm.

Representative histological images of the lumbar L4/L5 and L5/L6 IVDs from LBP-10X and sham rats are shown in **Figures 5A–D**. The histological grading scores (HGS) of the L4/L5 and L5/L6 IVDs are summarized in **Table 2**. In the sham group, the L4/L5 and L5/L6 IVDs showed a sharp boundary between the NP and AF at both the cranial/caudal and dorsal/ventral aspects of the disks, well-opposed AF lamellae and intact NP (**Figures 5A,C**). The median HGS for both the L4/L5 and L5/L6 IVDs for each of the parameters assessed was ‘0’ (**Table 2**). In contrast, in LBP-10X rats, the L4/L5 and L5/L6 IVDs exhibited loss of a distinct boundary between the AF and NP at both the cranial/caudal and dorsal/ventral aspects of the disks, poorly opposed AF lamellae with significant vertical tears, and degeneration of the NP (**Figures 5B,D**).

**Table 2:** Median IVD scores^∗^ for (x/y)^†^ based on histological findings for L4/L5 and L5/L6 IVDs of LBP-10X and sham-rats.

	Sham (*n* = 3)	LBP-10X (*n* = 5)
Loss of sharp boundary between NP and AF at cranial and caudal aspect of disk	0/0	2/2
Loss of sharp boundary between NP and AF at dorsal and ventral aspect of disk	0/0	2/2
Disruption of AF	0/0	2/2
Degeneration/disorganization of NP	0/0	1/1

*NP, nucleus pulposus; AF, annulus fibrosus. ^∗^0, No abnormalities detected; 1, Mild change; 2, Moderate or marked change. ^†^Score for L4/L5 IVDs is followed by score for L5/L6 IVDs.*

### Anti-hyperalgesic Efficacy of Clinically Available Analgesics in LBP-Rats

#### Morphine

**FIGURE 6:**
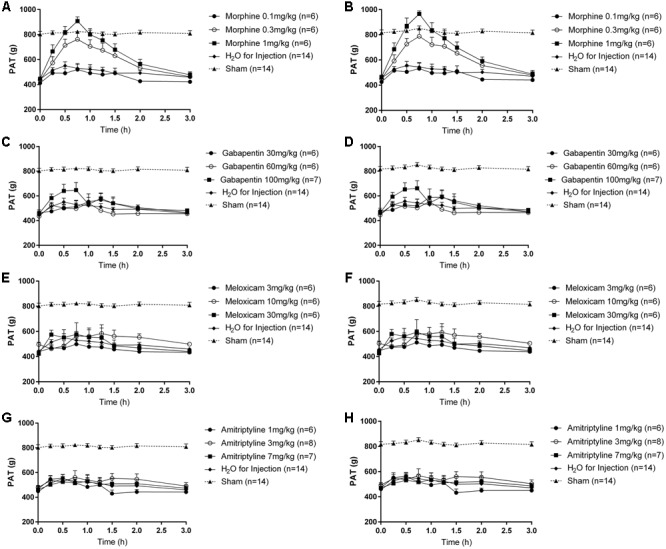
Temporal changes in the mean (±SEM) PAT values assessed in the lumbar axial deep tissues at **(A,C,E,G)** L1 and **(B,D,F,H)** L4/L5 in LBP-10X rats following administration of single subcutaneous bolus doses of **(A,B)** morphine (0.1, 0.3, and 1.0 mg/kg; *n* = 6 per dose), intraperitoneal (i.p.) bolus doses of **(C,D)** gabapentin (30, 60, and 100 mg/kg; *n* = 6–7 per dose), i.p. bolus doses of **(E,F)** meloxicam (3, 10, and 30 mg/kg; *n* = 6 per dose), i.p. bolus doses of **(G,H)** amitriptyline (1, 3, and 7 mg/kg; *n* = 6–8 per dose) or vehicle (*n* = 14). Single bolus doses of morphine, but not gabapentin, amitriptyline, meloxicam or vehicle, produced dose-dependent relief of mechanical hypersensitivity of lumbar axial deep tissues at L4/L5 (primary hyperalgesia) and L1 (secondary hyperalgesia). The dotted line in **(A–H)** indicates the mean PATs of sham rats.

In LBP-10X rats with fully developed primary and secondary mechanical hyperalgesia in the lumbar axial deep tissues at the L4/L5 and L1 regions, respectively, single s.c. bolus doses of morphine produced dose-dependent anti-hyperalgesia at both lumbar levels (**Figures 6A,B**), whereas vehicle was inactive. Morphine-evoked anti-hyperalgesia had a mean peak effect at 0.75 h post-dosing (**Figures 6A,B**). At the highest dose tested (1 mg/kg), the mean duration of action was approximately 3h (**Figures 6A,B**). The estimated mean ED_50_ values for morphine at L1 and L4/L5 were 0.48 (95% CI: 0.13 to 10.55) mg/kg and 0.46 (95% CI: 0.14 to 5.50) mg/kg, respectively. The mean ( ± SEM) ΔPAT AUC values for each dose of morphine administered to LBP-10X rats are summarized in **Table 3**.

**Table 3:** Dose-dependent extent and duration of anti-hyperalgesia [mean (±SEM) ΔPAT AUC values] evoked by single s.c. doses of morphine and single i.p. bolus doses of gabapentin, meloxicam and amitriptyline in LBP-10X rats.

Test compound and route of administration^∗^	Number of rats tested	Lumbar 1		Lumbar 4/5	
		Dose (mg/kg)	ΔPAT AUC	Dose (mg/kg)	ΔPAT AUC
Morphine (s.c.)	6	0.1	159.3 (43.5)	0.1	171.6 (44.8)
	6	0.3	477.5 (66.6)^¶^	0.3	461.9 (80.8)^¶^
	6	1	625.4 (87.7)^¶^	1	648.6 (85.5)^¶^
	14	Vehicle	15.8 (14.4)	Vehicle	57.03 (13.8)
Gabapentin (i.p.)	6	30	177.8 (81.5)	30	196 (90.5)
	6	60	128.1 (55.8)	60	126.4 (49.9)
	7	100	267.7 (66.1)	100	273.6 (73.4)
	14	Vehicle	62.4 (14.0)	Vehicle	57.6 (13.8)
Meloxicam (i.p.)	6	3	92.93 (43.6)	3	97.63 (48.0)
	6	10	188.7 (85.7)	10	193.9 (84.3)
	6	30	233.9 (99.7)	30	264.6 (100.2)
	14	Vehicle	65.35 (14.4)	Vehicle	57.03 (13.8)
Amitriptyline (i.p.)	6	1	83.95 (25.7)	1	98.49 (35.4)
	7	3	88.8 (44.4)	3	72.43 (36.7)
	6	7	62.66 (25.7)	7	58.6 (26.3)
	14	Vehicle	62.4 (14.0)	Vehicle	57.6 (13.8)

*^∗^s.c., subcutaneous; i.p., intraperitoneal; ^¶^*P* ≤ 0.05 (Kruskal–Wallis test, *post hoc* Dunn’s test) *c.f.* rats that received single bolus doses of vehicle.*

#### Gabapentin

Following administration of single bolus doses of gabapentin to LBP-10X rats, there was a trend for anti-hyperalgesia at the highest dose tested (100 mg/kg) (**Figures 6C,D**). But, this did not reach statistical significance as the mean (±SEM) ΔPAT AUC values were not significantly different (*P* > 0.05) from that for vehicle (**Table 3**).

#### Meloxicam and Amitriptyline

At the doses tested herein, single bolus doses of meloxicam (**Figures 6E,F**) and amitriptyline (**Figures 6G,H**) did not significantly alleviate (*P* > 0.05) primary or secondary mechanical hyperalgesia in the lumbar axial deep tissues at L4/L5 or L1 levels, respectively.

## Discussion

We have established and characterized a new rat model of chronic mechanical LBP using behavioral, pharmacologic and histologic methods. Importantly, our new LBP-model is devoid of a neuropathic component in contrast to most previously reported models (**Table 4**). Specifically, our findings show that induction of 5 or 10 small (0.5 mm outer diameter), shallow punctures (2 mm deep) to the L4/L5 and L5/L6 IVDs, without removal of the NP, resulted in temporal development of primary and secondary mechanical hyperalgesia in the lumbar axial deep tissues at the L4/L5 and L1 levels, respectively. Despite considerable morphologic changes induced in the punctured IVDs (**Table 2**), very good general animal health was maintained for the entire 49-day study duration. By inducing multiple small punctures of a consistent depth of only 2 mm to the lumbar IVDs, we avoided producing potentially confounding disk protrusions as evidenced by the absence of persistent mechanical allodynia in the hindpaws and no significant changes in gait for the 49-day study duration. Hence, contrary to previously reported rat models of chronic LBP (Hu and Xing, 1998; Tachihara et al., 2007; Gu et al., 2008; Amaya et al., 2009; Henry et al., 2012; Lai et al., 2015) that exhibited concurrent mechanical allodynia in the rat hindpaws (**Table 4**), our new model induced only mechanical hypersensitivity in the lumbar axial deep tissues at L4/L5 and L1 without a potentially confounding neuropathic component. These findings confirm our research hypothesis that it is possible to generate a rat model of chronic mechanical LBP that is devoid of disk herniation and any associated nerve root impingement.

**Table 4:** Comparison of the novel rat models (LBP-5X and LBP-10X) of chronic mechanical LBP with previously reported rodent models of LBP.

				Significant pain nocifensive behaviors					
Gender/species	Model induction	Study duration	Body weight changes	Mechanical allodynia	Cold allodynia	Pressure hyperalgesia^¶^	Thermal hyperalgesia	Gait changes	Reference
**OUR RAT MODEL OF MECHANICAL LBP**									
Male/SD	Annular puncture of L4/L5 and L5/L6 IVDs 5 times each using 25G needle (0.5 mm diameter, 2 mm deep)	49 days	No	No	NA	Yes	NA	NA	–
	Annular puncture of L4/L5 and L5/L6 IVDs 10 times each using 25G needle (0.5 mm diameter, 2 mm deep)	49 days	No	No	NA	Yes	NA	No	–
**PREVIOUS RODENT MODELS OF LBP**									
Male/SD	Application of CFA to the lumbar L4 spinal nerve	14 days	No	Yes	NA	NA	Yes	NA	Amaya et al., 2009
Male/SD	Lumbar L5/L6 facet joint compression injury	28 days	NR	Yes	NA	Yes	NA	NA	Henry et al., 2012
Male and Female/ SD	Chronic compression of L5 DRG	42 days	NR	NA	NA	NA	Yes	NA	Hu and Xing, 1998
Female/SD	Posterior puncture of L4/L5 IVD using 0.4 mm diameter needle, and removal of nucleus pulposus by injecting small amount of air	21 days	NR	NA	NA	NA	NA	NA	Olmarker, 2008
Male/SD	Annular puncture of L4/L5 and L5/L6 IVDs using a microsurgical drill of either (i) 0.5 mm diameter; 2 mm deep or (ii) 0.8 mm diameter; 2 mm deep and removal of the nucleus pulposus	49 days	NR	No	NA	Yes	NA	No	Kim et al., 2011
Male/SD	Midline puncture of L3/L4, L4/L5, and L5/L6 IVDs using a 26 g needle (3 mm deep) and injection of 2.5 μL of PBS	42 days	Minor reduction in LBP group	Yes	NA	NA	Yes	Yes	Lai et al., 2015
Male/SD	Posterior or Anterior annular puncture of L4/L5 IVD (21 g needle, 3 mm deep)	56 days	NR	Posterior: Yes Anterior: No	NA	NA	Posterior and Anterior: Yes; but minor	No	Li et al., 2014
Male/SD	Annular puncture of L3/L4, L4/L5 L5/L6 using 26G needle at either 1.5 mm (shallow) or 3 mm (deep) with injection of (i) PBS (ii) TNFα, (iii) NGF/VEGF	42 days	No	Yes for both shallow and deep punctures	NA	NA	NA	NA	Lai et al., 2016
Male/SPARC-null	Inactivation of SPARC IVD protein	78 weeks	NR	No	Yes	NA	No	NA	Millecamps et al., 2012

*^¶^Assessed using algometer. ^∗^NR, not reported; NA, not assessed; PBS, phosphate buffered saline; SD, Sprague-Dawley; TNF, tumor necrosis factor; NGF, nerve growth factor; VEGF, vascular endothelial growth factor.*

For our LBP-10X rats with 10 small, shallow, punctures in the L4/L5 and L5/L6 IVDs, the extent to which primary hyperalgesia developed in the lumbar axial deep tissues at L4/L5 was significantly greater (*P* ≤ 0.05) than that for LBP-5X rats with only 5 punctures in these IVDs. Owing to the small animal size and the narrow distance between the two punctured lumbar IVDs, it was not possible to assess mechanical hyperalgesia in the lumbar axial deep tissues at L5/L6 as well as at L4/L5 (Kim et al., 2011). Interestingly, there was concurrent temporal development of secondary hyperalgesia in the lumbar axial deep tissues at L1 in both LBP-5X and LBP-10X rats. These observations are potentially explained by the fact that the L4/L5 IVDs receive sensory nerve fiber innervations from the L1/L2 dorsal root ganglia (DRGs) in addition to innervations by sensory fibers from the DRGs at the corresponding level (L4/L5) (Ohtori et al., 1999, 2001; Chen et al., 2008). It is also possible that application of the pressure algometer to the lumbar axial deep tissues at L1 may have produced larger bending moments at the levels of L4/L5 or L5/L6 where there is greater irritation due to the induced disk lesions (Henry et al., 2012). Our findings are aligned with previous work by others that showed similar reductions in noxious pressure thresholds applied at various lumbar levels following annular puncture of lumbar IVDs or lumbar facet joint compression injury (Kim et al., 2011; Henry et al., 2012).

In humans, chronic mechanical or discogenic LBP is the leading cause of disability globally, due to a lifetime prevalence of 50–90% (Hsu et al., 2015). It is also associated with enormous socioeconomic and personal costs to the affected individuals, their families and society (Hsu et al., 2015). Rodent models that more closely mimic individual human pain conditions are invaluable for gaining new insight on the specific pathobiology underpinning each condition. These models are also useful for screening novel compounds from drug discovery to identify those with potential to be highly efficacious and well-tolerated novel analgesics and/or adjuvants (Muralidharan et al., 2013). Previously reported rodent models of chronic LBP where there is prominent development of mechanical allodynia in the hindpaws (Hu and Xing, 1998; Tachihara et al., 2007; Gu et al., 2008; Amaya et al., 2009; Henry et al., 2012; Lai et al., 2015) akin to lumbar radicular pain in humans (Defrin et al., 2014), have been critically questioned (Devor and Tal, 2009). This is because typical patients with chronic mechanical LBP do not report prominent neuropathic pain symptoms in the lower extremities (Devor and Tal, 2009; Defrin et al., 2014).

In the present study in rats with either 5 or 10 small, shallow (2 mm deep) punctures in the L4/L5 and L5/L6 IVDs, mechanical allodynia, a hallmark symptom of lumbar radicular pain (Devor and Tal, 2009; Defrin et al., 2014), did not develop, in contrast to multiple LBP-models reported by others (**Table 4**). In rats where the NP was removed from the L4/L5 and L5/L6 IVDs (Kim et al., 2011) or involving induction of a single large puncture wound [21 G needle (i.e., 0.82 mm outer diameter), 3mm deep] in the anterior surface of the L4/L5 IVDs (Li et al., 2014), mechanical hyperalgesia developed in the lumbar axial deep tissues at L4/L5 and/or hindpaw hypersensitivity was absent.

In LBP-10X rats, histologic assessment of the L4/L5 and L5/L6 IVDs showed significant morphologic changes indicative of IVD degeneration. Specifically, the L4/L5 and L5/L6 IVDs of LBP-10X rats exhibited loss of a sharp boundary between the NP and AF at both the cranial/caudal and dorsal/ventral aspects of the disks, poorly opposed AF lamellae with significant vertical tears and degeneration of the NP. These findings are similar to the morphological changes observed in cadaveric and surgical human IVD specimens, collected from both symptomatic and asymptomatic individuals, showing AF lamellar disorganization and fissures, as well as a disorganized and/or degenerated NP region (Thompson et al., 1990; Boos et al., 2002; Roberts et al., 2006; Vernon-Roberts et al., 2007, 2008; Freemont, 2009; Weiler et al., 2011; Rutges et al., 2013). This suggests segmental instability of the lumbar spine of LBP-10X rats and may potentially result in biomechanical and/or biological abnormalities of the IVDs. Our LBP-10X model is an advance in the field as it closely mimics chronic mechanical LBP in humans where there are morphological and structural changes in disks, suggestive of reduced structural integrity of the IVDs.

There are well-documented changes in gait parameters in rodent models of neuropathic pain (Bozkurt et al., 2008; Huehnchen et al., 2013; Lalli et al., 2013) and in humans affected by LBP with a radicular component (Shamji et al., 2009; Huang et al., 2011; Seay et al., 2011; Simmonds et al., 2012; Miyagi et al., 2013; Muller et al., 2015). In our present work, the absence of significant (*P* > 0.05) temporal changes in the gait parameter of inter-limb coordination, underscores the absence of a lumbar radicular component in our new rat model of mechanical LBP. This latter finding further highlights that our new rat model more closely mimics that of patients with chronic mechanical LBP in the clinical setting than previous models (**Table 4**).

In LBP-10X rats, single s.c. bolus doses of morphine produced dose-dependent relief of primary and secondary mechanical hyperalgesia in the lumbar axial deep tissues at L4/L5 and L1, respectively, whereas vehicle was inactive. Although there was a trend for gabapentin to produce a brief period of partial pain relief at the highest dose (100 mg/kg) tested, this response did not differ significantly (*P* > 0.05) from that evoked by vehicle. The doses of gabapentin were not escalated further to avoid potentially confounding sedation. Neither amitriptyline nor meloxicam evoked significant anti-hyperalgesia in LBP-10X rats Importantly, the absence of a neuropathic component in our rat model of LBP is further supported by the lack of efficacy of both gabapentin and amitriptyline, medications with proven efficacy in neuropathic pain conditions (Abdi et al., 1998). In work by others using a more severe rat model of LBP involving removal of the NP from the lumbar disks at L4/L5 and L5/L6, oral pregabalin at 20 mg/kg lacked efficacy for the relief of primary mechanical hyperalgesia in the lumbar axial deep tissues (Kim et al., 2011). Gabapentin and pregabalin reportedly lack efficacy for relief of chronic mechanical LBP in patients (Atkinson et al., 2010; Sakai et al., 2015). Herein, gabapentin produced partial relief of primary and secondary mechanical hyperalgesia in the lumbar axial deep tissues at L1 and L4/L5 in LBP-10X rats. In work by others using a rat model of IVD injury, the extracellular glutamate concentration was markedly increased due to enzymatic breakdown of aggrecan in the disk cartilage extracellular matrix, and slow clearance of glutamate due the avascular nature of IVDs and their low rates of cellular metabolism (Osgood et al., 2010). A pronociceptive role for this IVD glutamate ‘reservoir’ may be underpinned by upregulated expression levels of glutamate receptors (NMDA and AMPA) in the lumbar DRGs (Osgood et al., 2010). Whether expression levels of the α_2_δ_1_ subunit of voltage-gated calcium channels in the lumbar DRGs and/or the spinal dorsal horn are also increased (Kim et al., 2011), remains for future investigation in our new model.

## Conclusion

We have successfully established a new rat model of chronic mechanical LBP by induction of either 5 (LBP-5X) or 10 (LBP-10X) minor mechanical insults to two adjacent lumbar IVDs and we characterized our new model using behavioral, histologic and pharmacologic methods. Our LBP-10X model is a significant advance over many other rodent models of chronic LBP as animals exhibit primary and secondary mechanical hyperalgesia in the lumbar axial deep tissues at L4/L5 and L1, respectively, with the NP remaining intact during the model induction procedure. Importantly in our model, mechanical allodynia did not develop in the hindpaws and there were no significant changes in gait. Our new rat model of LBP has the potential to provide novel insight on the pathobiology of this disabling chronic pain condition in humans. It also has applicability for efficacy profiling of novel compounds aimed at discovery of new and well-tolerated analgesics for improved relief of chronic mechanical LBP in humans.

## Author Contributions

MS and AM designed the studies described herein. AM performed the model establishment and optimization experiments. TP performed the histology and pharmacology experiments. JM, a board certified Diplomate of the American College of Veterinary Pathologists, analyzed and interpreted the histology data. LG and AK performed the gait analysis experiments. JN and LC contributed substantial knowledge and insights that were helpful to the design of the experiments, pertinent comments on the data generated and critiqued manuscript drafts. AM and MS wrote the manuscript. All authors reviewed manuscript drafts and approved the final manuscript.

## Conflict of Interest Statement

The authors declare that the research was conducted in the absence of any commercial or financial relationships that could be construed as a potential conflict of interest. The reviewer CP and handling Editor declared their shared affiliation, and the handling Editor states that the process met the standards of a fair and objective review.
